# Supplementary material for: Molecular Pro-Apoptotic Activities of Flavanone Derivatives in Cyclodextrin Complexes: New Implications for Anticancer Therapy
Source: Int J Mol Sci. 2024 Aug 3;25(15):8488. doi: 10.3390/ijms25158488 (PMC11312998; doi:10.3390/ijms25158488)
Supplement: Supplementary file 1 [file ijms-25-08488-s001.zip › ijms-3077800-supplementary.pdf]

Molecular Pro-apoptotic Activities of Flavanone Derivatives in Cyclodextrin Complexes: New Implications for Anticancer Therapy

Angelika A. Adamus-Grabicka,[1] Paweł Hikisz[2], Artur Stepniak[3], Magdalena Malecka[3], Piotr Paneth[4], Joanna Sikora[1], Elzbieta Budzisz[5]

[1]Department of Bioinorganic Chemistry, Faculty of Pharmacy, Medical University of Lodz, Muszynskiego 1, Lodz, 90-151, Poland angelika.adamus@umed.lodz.pl
[2]Department of Molecular Biophysics, Faculty of Biology and Environmental Protection, University of Lodz, Pomorska 141/143, 90-236 Lodz, Poland pawel.hikisz@biol.uni.lodz.pl
[3]Department of Physical Chemistry, Faculty of Chemistry, University of Lodz, Pomorska 163/165, Lodz, 90-236, Poland artur.stepniak@chemia.uni.lodz.pl; magdalena.malecka@chemia.uni.lodz.pl
[4]Institute of Applied Radiation Chemistry, Lodz University of Technology, Zeromskiego 116, 90-924 Lodz piotr.paneth@p.lodz.pl
[5]Department of the Chemistry of Cosmetic Raw Materials, Medical University of Lodz, 90-151 Lodz, Poland, elzbieta.budzisz@umed.lodz.pl

S1. The effect of the tested compounds (**3**; **5**, **β-CD** and the combinations **β-CD+3** and **β-CD+5**) in the concentration range of 1-100 μmol/L on damage to the protein-lipid membrane of RBCs, expressed as % hemolysis. There is a statistically significant difference compared to the control * - p=0.05; ** - p = 0.01 - 0.001. Results are presented as mean values ± standard deviation (SD) from three independent experiments.

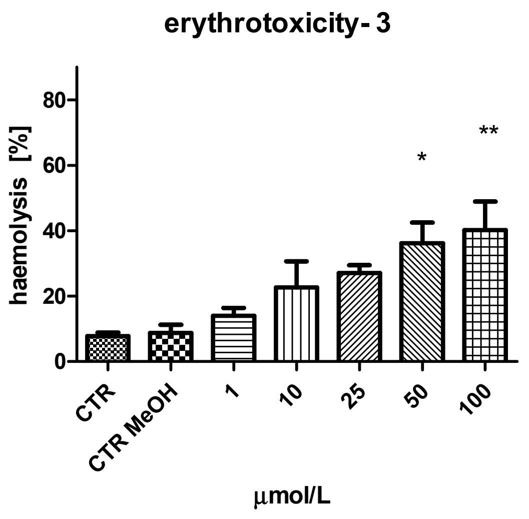
erythrotoxicity- 3
haemolysis [%]
0
20
40
60
80
*
**
CTR
CTR MeOH
1
10
25
50
100
μmol/L

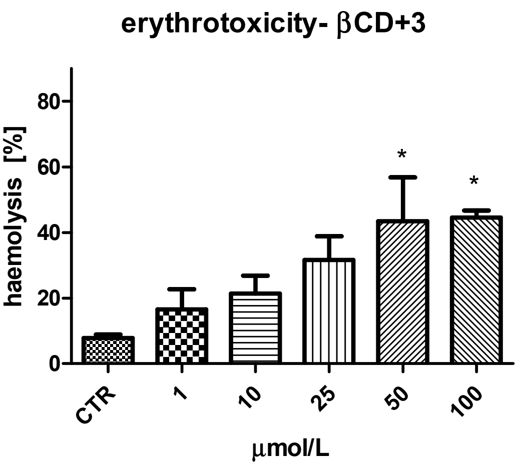
erythrotoxicity- βCD+3
haemolysis [%]
0
20
40
60
80
*
*
CTR
1
10
25
50
100
μmol/L

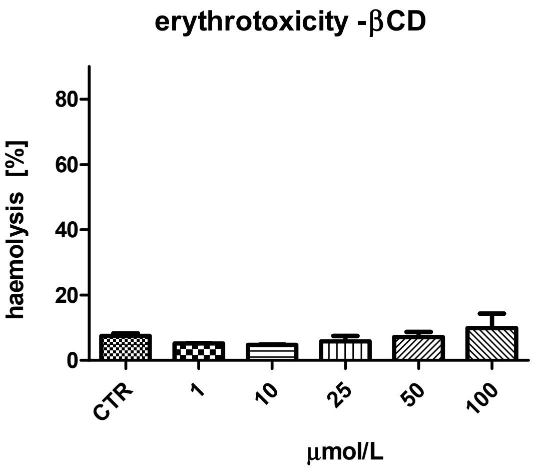
erythrotoxicity -βCD
haemolysis [%]
0
20
40
60
80
CTR
1
10
25
50
100
μmol/L

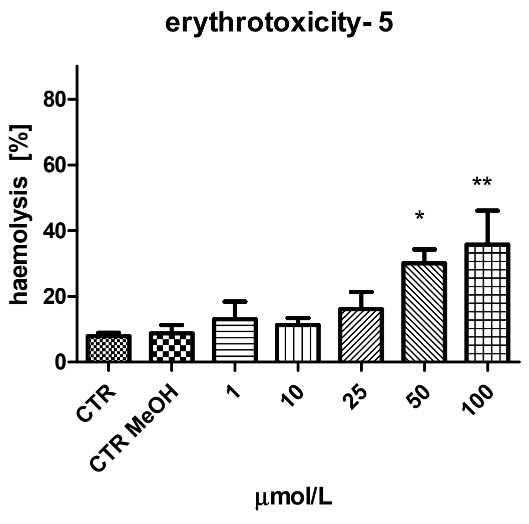
erythrotoxicity- 5
haemolysis [%]
0
20
40
60
80
*
**
CTR
CTR MeOH
1
10
25
50
100
μmol/L

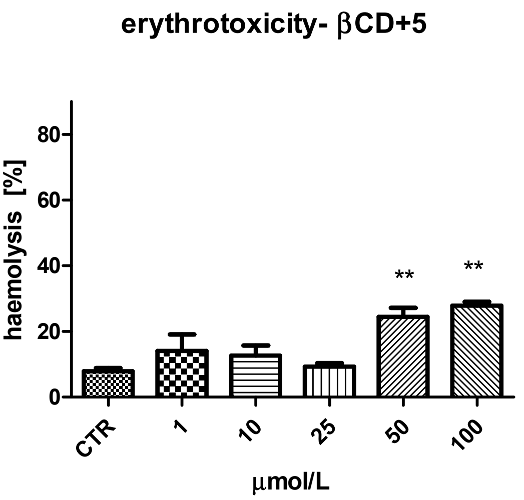
erythrotoxicity- βCD+5
haemolysis [%]
0
20
40
60
80
**
**
CTR
1
10
25
50
100
μmol/L
